# Observing GLUT4 Translocation in Live L6 Cells Using Quantum Dots

**DOI:** 10.3390/s110202077

**Published:** 2011-02-10

**Authors:** Feng Qu, Zubin Chen, Xiaoxuan Wang, Lingfeng Meng, Zhengxing Wu, Anlian Qu

**Affiliations:** Institute of Biophysics and Biochemistry, School of Life Science and Technology, Huazhong University of Science and Technology, Wuhan 430074, China; E-Mails: phonequ@gmail.com (F.Q.); chenzubinczb@126.com (Z.C.); af_wxx@163.com (X.W.); menglingfeng.xy@163.com (L.M.); ibbwuzx@mail.hust.edu.cn (Z.W.)

**Keywords:** quantum dots, GLUT4, confocal microscopy, translocation, endocytosis

## Abstract

The glucose transporter 4 (GLUT4) plays a key role in maintaining whole body glucose homeostasis. Tracking GLUT4 in space and time can provide new insights for understanding the mechanisms of insulin-regulated GLUT4 translocation. Organic dyes and fluorescent proteins were used in previous studies for investigating the traffic of GLUT4 in skeletal muscle cells and adipocytes. Because of their relative weak fluorescent signal against strong cellular autofluorescence background and their fast photobleaching rate, most studies only focused on particular segments of GLUT4 traffic. In this study, we have developed a new method for observing the translocation of GLUT4 targeted with photostable and bright quantum dots (QDs) in live L6 cells. QDs were targeted to GLUT4myc specifically and internalized with GLUT4myc through receptor-mediated endocytosis. Compared with traditional fluorescence dyes and fluorescent proteins, QDs with high brightness and extremely photostability are suitable for long-term single particle tracking, so individual GLUT4-QD complex can be easily detected and tracked for long periods of time. This newly described method will be a powerful tool for observing the translocation of GLUT4 in live L6 cells.

## 1. Introduction

Glucose transporter 4 (GLUT4), the main glucose transporter activated by insulin in skeletal muscle cells and adipocytes, plays a key role in maintaining whole body glucose homeostasis [1–3]. Previous studies have provided evidences that externalization of GLUT4 is defective in the pathophysiological state of insulin resistance underlying type II diabetes [4–6], so it is valuable to research mechanism of GLUT4 translocation in live cells to uncover the nosogenesis of this disease. Under basal condition, about 3–10% of the GLUT4 is located at the cell surface and more than 90% is in intracellular compartments [7]. Upon insulin stimulation, approximately 50% of the GLUT4 is rapidly recruited to the plasma membrane (PM) by significantly enhancing their exocytosis and minimally reducing their endocytosis [8]. In the absence of insulin, GLUT4 constitutively cycles to and from the PM, and insulin sharply increases the rate of GLUT4 recycling [9,10]. However, more details of the molecular mechanism of intracellular GLUT4 translocation in insulin-stimulated cells are required. Tracking GLUT4 molecules in space and time might provide new evidences to understanding the mechanisms of insulin-regulated GLUT4 translocation.

In previous studies organic dyes and fluorescent proteins, such as Texas Red, Cy3 and EGFP, were used to observe GLUT4 translocation in live cells [11–14]. However, these studies only observed particular segments of GLUT4 traffic due to their rapid photobleaching and relative weak fluorescent signal against strong cellular autofluorescence background. Quantum dots (QDs) are protein-sized crystals of inorganic semiconductors composed of atoms from groups II–VI or III–V elements in the periodic table [15,16]. Compared with organic dyes and fluorescent proteins, QDs offer several unique advantages, such as size-tunable emission from visible to infrared wavelengths, a broad absorption spectrum, a narrow emission spectrum, very high levels of brightness and photostability [17–20]. QDs coupled with biorecognition molecules such as streptavidin, peptides, proteins, and DNA [21–24], overcome the limitations that conventional dyes suffer from, and provide useful alternatives for long-term multicolor cellular, molecular, and *in vivo* imaging [21,25,26]. Thus, labeling of GLUT4 in live cells with QDs can provide a new insight into GLUT4 translocation mechanisms.

To label and image GLUT4 in live cells with QDs, we have developed a novel assay based on L6 cells [27], a typical model system for investigating the mechanism of GLUT4 translocation in skeletal muscles [11,28]. However, neither exocytosis nor endocytosis of GLUT4 vesicles could be investigated after this labeling procedure. Because GLUT4 was dynamically labeled with QDs as it cycled between intracellular compartments and the PM, it was difficult to recognize which GLUT4-QD was translocated from intracellular compartments to PM and which GLUT4-QD was internalized from PM to intracellular compartments. In recent research by Fujita, a QD-based analysis of insulin-stimulated GLUT4 trafficking processes in fully differentiated 3T3L1 adipocytes was performed [29]. However, dynamic processes of GLUT4 endocytosis and recycle cannot be observed. In this study, we have labeled L6 cells with IgG conjugated QDs in 4 °C. The experimental time was reduced by using QD-IgG conjugates. The GLUT4-QD complex was located on PM and internalization of GLUT4 can be observed after this simplified labeling procedure. Coupled with Andor Revolution XD laser confocal microscope system, three dimensional trajectory of GLUT4 in live L6 cells can be observed clearly in real time.

## 2. Materials and Methods

### 2.1. Materials

Anti-c-myc monoclonal antibody 9E10 and Qdot 605 goat anti-mouse IgG conjugate were purchased from Invitrogen. L6-GLUT4myc cells were provided by Prof. Amira Klip, the Hospital for Sick Children (Toronto, Ontario, Canada). GLUT4-EGFP plasmid was provided by Prof. Tao Xu (Institute of Biophysics of the Chinese Academy of Sciences, Beijing, China). Other chemicals were purchased from Invitrogen.

### 2.2. Cell Culture

L6-GLUT4myc myoblasts were cultured in α-MEM supplemented with 10% fetal bovine serum and 1% antibiotic at 37 with 5% CO_2_.

### 2.3. Labeling GLUT4myc Molecular with QDs

L6-GLUT4myc cells were seeded in growth medium (α-MEM, 10% fetal bovine serum, and 1% antibiotic) at 30–40% confluence the previous day. They were starved with depletion medium (α-MEM and 1% antibiotic) for 3 h at 37 °C prior to experimentation to enhance the effect of insulin stimulation. Then, they were stimulated with 100 nM insulin at 37 °C for 20 min. Later, they were blocked with 5% goat serum in α-MEM containing 100 nM insulin for 10 min at 37 °C and then incubated with anti-myc monoclonal antibody 9E10 (with an initial concentration of 0.5 mg/mL, 1:100 diluted in α-MEM) containing 100 nM insulin for 1 h at 37 °C. Unbound antibody was aspirated away and the cover slips were washed three times with ice-cold α-MEM containing 100 nM insulin at 4 °C. Later, they were blocked with 5% goat serum in α-MEM containing 100 nM insulin for 10 min at 4 °C and incubated with Qdot 605 goat anti-mouse IgG conjugate (with an initial concentration of 1 μM, 1:50 diluted in α-MEM) containing 100 nM insulin for 1 h at 4 °C. Unbound Qdot-IgG was aspirated away and the cover slips were washed five times with ice-cold α-MEM containing 100 nM insulin at 4 °C. Finally, the cells were cultured in growth medium at 37 °C for imaging. For control, two groups were prepared. One group was stimulated with insulin and then incubated with QD-IgG diluted in growth medium containing 100 nM insulin for 1 h at 4 °C, the primary antibody 9E10 was omitted during the labeling procedure in this group. Another group was not stimulated with insulin and insulin was absent throughout the labeling procedure.

### 2.4. Transfection with GLUT4-EGFP

L6-GLUT4myc cells were seeded in growth medium (α-MEM, 10% fetal bovine serum, and 1% antibiotic) at 80% confluence prior to experimentation. For each chamber, 0.8 μg GLUT4-EGFP plasmid was diluted into 50 μL OMEM without fetal bovine serum. Simultaneously, 2 μL Lipofectamine™ 2000 was diluted to 50 μL OMEM without fetal bovine serum. After 5 min, GLUT4-EGFP and Lipofectamine™ 2000 were combined within 20 min. The cells were cultured at 37 °C for 4 h after the complexes were added directly and mixed gently. Finally, the cells were cultured in growth medium at 37 °C and ready to image after 20 h post transfection.

### 2.5. Optical System and Image Processing

The optical system for two-dimensional observation of GLUT4-QDs distribution consisted of an inverted wide-field microscope (Axiovert X100 TV, Zeiss), a monochromatic light source (Polychrome IV, TILL), a 100× oil objective lens (UplanApo, 100×, 1.35 NA, Olympus) and a cooled digital CCD (sensicam qe, PCO). The excitation wavelength was 488nm and the fluorescence from QDs (605 nm) was filtered by a >600 nm long pass filter. The expose time was 200 ms. Images were preprocessed by TILLvisION software.

A laser-based spinning disk confocal microscopy system (Revolution XD, ANDOR) was used for observing three-dimensional distribution and dynamics of GLUT4-QDs in live L6 cells. A 60× objective lens was used and QDs were excited by a blue laser (491 nm). The laser power was 100%, and the fluorescence from QDs (605 nm) was filtered by a 610 ± 25 nm band pass filter and recorded by an EMCCD (DV885, ANDOR). For temporal series of three-dimensional images, a z-scanning from top to bottom with a 100 nm step was performed every minute. Images were taken and preprocessed by Andor iQ software.

Fluorescence images were processed and analyzed by ImageJ and MATLAB. Three-dimensional reconstruction was performed using Amira software (http://www.amiravis.com/).

### 2.6. Trajectory Analysis of GLUT4-QDs

L6-GLUT4myc cells were labeled with QDs as above, and then cultured in growth medium pre-warmed at 37 °C and transferred to a laser-based spinning disk confocal microscopy system for z-scanning. 4D (3D + t) fluorescence image stacks of live L6 cells were processed and analyzed by MATLAB. There were more than one GLUT4 protein in one GLUT4 storage vesicle, and the diameter of these vesicles was 50 nm [30]. It is likely that one vesicle contains more than one GLUT4-QD complex and these GLUT4-QDs are tracked together as one QD signal. The peak intensity position of a QD in three-dimensional was detected as the position of this QD. The position of nearest QD in 3D spatial at the next time point was determined as the next position of the QD, and the positions at different time point were connected in this way. The 3D spatial distances between the positions at different time moments and the position at initial moment were calculated by the following equation:

$$S_{n}=\sqrt{{\left(x_{n}-x_{0}\right)}^{2}+{\left(y_{n}-y_{0}\right)}^{2}+{\left(z_{n}-z_{0}\right)}^{2}}$$

where x_n_, y_n_ and z_n_ are positions on frame n. The velocity of GLUT4-QDs averaged for the period of 1 min was calculated by the following equation:

$$v_{n}=\sqrt{{\left(x_{n}-x_{n-1}\right)}^{2}+{\left(y_{n}-y_{n-1}\right)}^{2}+{\left(z_{n}-z_{n-1}\right)}^{2}}/\Delta t$$

where x_n_, y_n_ and z_n_ are positions on frame n, and Δt is the time interval between two frames.

## 3. Results and Discussion

GLUT4 continually cycles between intracellular compartments and the PM in basal state and stimulation state. In basal state, half-maximal recycling of GLUT4 is approximately 2 h, whereas insulin sharply increase the rate of GLUT4 recycling, reducing the halftime to about 40 min. All the intracellular GLUT4 molecules of L6 muscle cells recycle to the PM within 6 h in basal state, insulin accelerates this recycling so that all GLUT4 molecules recycle to the PM within 3 h [9]. Upon insulin stimulation, the rate of GLUT4 internalization is decreased by 70%, and the rate of GLUT4 translocation to the PM is sped up by 7- to 10-fold [31]. Insulin increases not only the amount of GLUT4 on the PM but also the average exposure time of GLUT4 on the cell surface. To label GLUT4 molecules on the outside surface of cell membranes, Klip and her coworkers have established a L6-GLUT4myc cell line with a myc epitope in the first exofacial loop [11,28,32]. GLUT4myc molecules were labeled by primary antibody as it cycled between the intracellular compartments and the PM in the presence of insulin, most GLUT4myc molecules could be labeled by primary antibody. Cells were transferred to 4 °C to halt all vesicular traffic and then incubated in the presence of insulin with QD-IgG. As a result, most GLUT4myc molecules on the PM were labeled by QDs and then internalized with QDs at 37 °C after insulin withdrawal.

### 3.1. The Specificity of the Labeling Protocol

**Figure 1.**
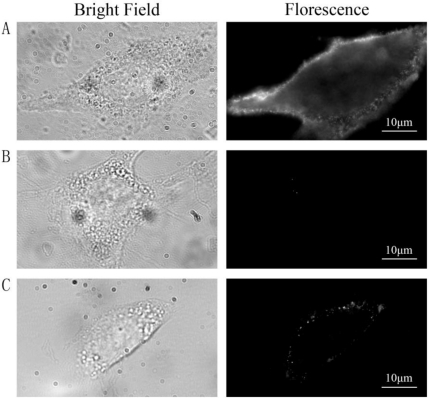
The specificity of the labeling protocol. **(A)** Group A was serum-starved and stimulated with 100 nM insulin, followed by labeling as described in the method. **(B)** Group B was stimulated with insulin and then incubated with QD-IgG diluted in growth medium containing 100 nM insulin for 1 h at 4 °C. **(C)** Group C was not stimulated with insulin and insulin was absent throughout the labeling procedure. QDs were located on the PM in group A indicated that GLUT4myc was labeled by QDs on the PM after labeling procedure. Almost no QDs appeared in the control group B indicating that QDs labeled GLUT4myc by antibody-antigen reaction, this protocol was specific. Fluorescence spots in group C were obviously less than those in group A indicating that insulin increased the rate of GLUT4myc recycling. The imaging parameters were identical across the different groups.

Three groups of cells were prepared and treated as follows: group A was labeled with QDs as described in the method, group B was stimulated with insulin and then incubated with QD-IgG diluted in growth medium containing 100 nM insulin for 1 h at 4 °C, and group C was not stimulated with insulin and insulin was absent throughout the labeling procedure. Images were acquired using wide-field microscope after labeling procedure. QDs were located on the PM in group A [Figure 1(A)] indicating that most GLUT4myc molecules on the PM were labeled by primary antibody at 37 °C, then GLUT4myc was stopped on the PM and labeled by QDs-IgG at 4 °C. In group B, almost no fluorescence spots were observed in the control cells [Figure 1(B)] suggesting that QDs detected in group A were specifically bound with GLUT4myc by antibody-antigen reaction and almost no QDs were bound on PM by nonspecific binding. In group C [Figure 1(C)], fluorescence spots were obviously less than those in group A, which indicated that insulin increased the rate of GLUT4myc recycling. These results illustrate that the labeling protocol is highly specific.

### 3.2. Internalization of the GLUT4-QD Complex

L6-GLUT4myc cells were labeled with QDs as described above, and then cultured in growth medium pre-warmed at 37 °C and transferred to a laser-based spinning disk confocal microscopy system for observing. We defined this time point as the beginning.

**Figure 2.**
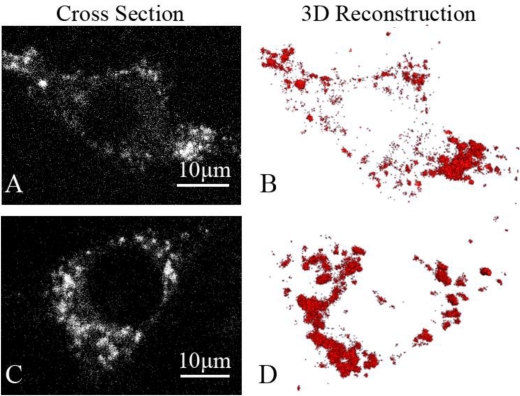
Three dimensional reconstruction of GLUT4-QD complex in L6 cell. L6-GLUT4myc cells were labeled with QDs as described in the method and fluorescence three-dimensional slices were acquired with a confocal microscopy system. **(A)** Cross section of the L6 cell at the beginning. **(B)** Three dimensional reconstruction of GLUT4-QDs at the beginning shown that GLUT4-QDs were located on the PM. **(C)** Cross section of the L6 cell after 12 h. **(D)** Three dimensional reconstruction of GLUT4-QDs after 12 h shown that GLUT4-QDs were internalized by endocytosis and clustered together round a conical perinuclear area. The imaging parameters were identical across the different panels.

**Figure 3.**
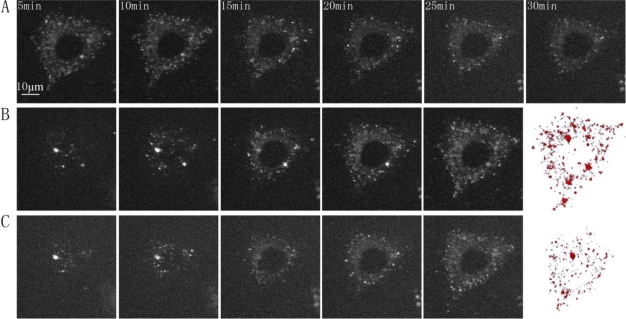
Internalization of GLUT4-QD complex in L6 cell. **(A)** Cross section of the same slice in a L6 cell at different times during internalization. The cross-section images of different layers in the L6 cell and three dimensional reconstruction of GLUT4-QD at the beginning **(B)** and 20 min later **(C)** show the change of the three-dimensional distribution of the GLUT4-QDs in the live L6 cell. The imaging parameters were identical across the different panels.

Fluorescence three-dimensional slices were acquired from z-scanning with a laser-based spinning disk confocal microscopy system at 37 °C [Figure 2(A)]. To further confirm the presence of the GLUT4-QDs in L6 cells, a three-dimensional reconstruction of the fluorescence three-dimensional slices was performed using Amira software [Figure 2(B)]. To observe the dynamic process of GLUT4-QDs internalization, a z-scanning over the whole cell was performed per minute for a period of 30 min. The cross sections of the cell indicated that GLUT4-QDs were internalized by the L6-GLUT4myc cells [Figure 3(A)] in 20 min, which is consistent with previous study [9]. The cross-section images of different layers in the L6 cell and the three-dimensional reconstructions of GLUT4-QDs at the beginning [Figure 3(B)] and 20 min later [Figure 3(C)] showed the change of the three-dimensional distribution of the LUT4-QDs in live L6-GLUT4myc cells. To investigate three-dimensional distribution of internalized GLUT4-QDs, fluorescence three-dimensional slices were acquired 12 h later. As shown in Figure 2(C) and Figure 2(D), the internalized GLUT4-QDs were distributed to a conical perinuclear area, which is highly consistent with previous studies [2,7]. These results indicated that QDs bound to and internalized with GLUT4.

### 3.3. Comparison of the Fluorescence Images between GLUT4-QDs and GLUT4-EGFP

**Figure 4.**
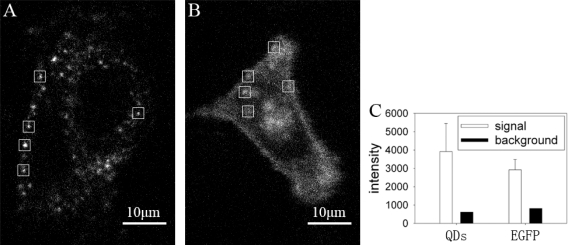
Comparison of the fluorescence images between GLUT4-QDs and GLUT4-EGFP. GLUT4 labeled with QDs and EGFP as described in method, and imaged under confocal microscopy. **(A)** Fluorescence image of GLUT4-QDs, exposure time was 100 ms. **(B)** Fluorescence image of GLUT4-EGFP, exposure time was 200 ms. Other imaging parameters were identical across this two fluoroscopic image. **(C)** Fluorescence intensity analysis of the five 20 × 20 pixel rectangle windows in (A) and (B). White bars represent the mean signal intensity and the lines above white bars represent the difference of mean signal intensity and maximum signal intensity. Black bars represent the background intensity.

We compared the fluorescence images of GLUT4-QDs with GLUT4-EGFP, which is a fluorescence protein widely used in the study of GLUT4 translocation. The fluorescence images were acquired under confocal microscope, excitation wavelength was identical (491 nm), and exposure time of EMCCD was 100 ms for QDs and 200 ms for EGFP. The fluorescence intensity of each image was analyzed with MATLAB. The fluorescence intensity of QDs was brighter than EGFP even though the exposure time of EGFP was twice as that of QDs. As shown in Figure 4(A) and Figure 4(B), single fluorescence particle of QD can be detected easier than that of EGFP. Fluorescence intensities of five 20 × 20 pixel rectangle windows in Figure 4(A) and Figure 4(B) were analyzed in Figure 4(C). The mean signal intensity of rectangle windows in Figure 4(A) was stronger than that in Figure 4(B), and the background intensity of rectangle windows in Figure 4(A) was lower than that in Figure 4(B). The QDs signal intensity in the rectangle windows of Figure 4(A) was 6 times stronger than background intensity, whereas the EGFP signal intensity in the rectangle windows of Figure 4(B) was 3.5 times stronger than background intensity. The ratio of QDs signal intensity and background intensity was significant higher than the ratio of EGFP signal intensity and background intensity (P < 0.05). As a result, it is easier to locate GLUT4 in live L6 cells by labeling with QDs. According to the needs of experiments, amount of GLUT4-QDs can be adjusted by changing reaction time and concentration of QD-IgG dilution to make GLUT4-QD easier to be detected, whereas GLUT4-EGFP can not. These results proved that QDs are more suitable for observing intracellular GLUT4 translocation than fluorescent proteins in live cells.

### 3.4. Trajectory Analysis of Individual GLUT4-QD in Three-Dimensions

**Figure 5.**
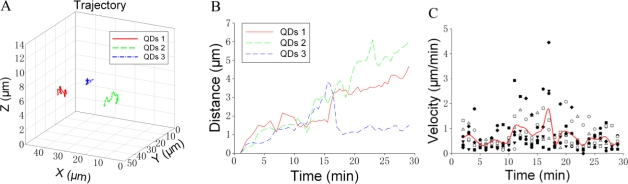
Trajectory analysis for GLUT4-QDs. **(A)** Trajectories of three GLUT4-QDs in a L6 cell. **(B)** Spatial distances between the positions at different time moments and the position at initial moment of three GLUT4-QDs. Two GLUT4-QDs moved far away from the initial position and one GLUT4-QD moved toward the initial position at about 15 min after endocytosis. **(C)** Velocities of seven different GLUT4-QDs averaged for the period of 1 min (dots), and mean value of these seven velocities (solid line). The velocities of these seven GLUT4-QDs had significant changes at about 10 min to 20 min of internalization.

We attempted to analyze the trajectory of individual GLUT4-QD in three-dimensions. Figure 5 shows an example of the trajectory analysis for GLUT4-QDs. The trajectories of three GLUT4-QDs in a live L6 cell were shown in Figure 5(A). To analyze the trajectories of these GLUT4-QDs, we calculated the spatial distances, which between the positions at different time moments and the position at initial moment [Figure 5(B)]. Furthermore, the velocity of GLUT4-QDs averaged for the period of 1 min was calculated. As shown in Figure 5(C), different types of dots represent the velocities of seven GLUT4-QDs, and solid line represents the mean value of these seven velocities. Additionally, we divided the velocities of these seven different GLUT4-QDs into three groups (Group A: 0–10 min, Group B: 10–20 min and Group C: 20–30 min), and then compared the mean velocities between these three groups using the Tukey Test. There are statistically significant differences between Group B and other two groups (P < 0.05), and there is statistically no difference between Group A and Group C. It demonstrated that the velocity of these seven GLUT4-QDs had significant changes at about 10 min to 20 min of internalization, some GLUT4-QDs moved far away from the initial position and some GLUT4-QDs moved toward the initial position at this time period. This result is consistent with previous study, which has proposed a model of intracellular GLUT4 traffic [9]. In this model, internalized GLUT4 travels through the early endosome in 5 min and progresses to the recycling endosome in 20 min in the absence of insulin; at the same time, a fraction of the internalized GLUT4 transport directly from early endosome to the specialized vesicles pool. From what has been discussed above, this method is suitable for observing GLUT4 translocation and can provide visual evidences for GLUT4 internalization study.

We attempted to provide a specific and long-term imaging platform for observing GLUT4 traffic mechanisms and the interactions between GLUT4 vesicles and cellular organelles. In live cells, tracking single molecules labeled with either fluorescent proteins or organic dyes remains a challenging task, because of their relative weak fluorescent signal against strong cellular autofluorescence background and their fast photobleaching rate [33]. Recent studies have used QDs as probes in single molecule tracking experiments, but most of them were labeling QDs on membrane receptors or membrane associated proteins [34–37], because they do not require intracellular delivery through the impermeable plasma membrane. To labeling intracellular motor proteins by QDs, previous works attempted to use membrane translocation peptides or transfection agents, single cell microinjection and endocytosis [26,38–40]. Previous works of our lab have introduced QDs into the research of the mechanism of GLUT4 traffic [27]. GLUT4 was dynamically labeled with QDs as it cycled between intracellular compartments and the PM. After labeling procedure, most QDs were internalized with GLUT4, and the complexes of GLUT4-QD were in different segments of translocation, so it was difficult to distinguish exocytosis from endocytosis of GLUT4-QD vesicles. Furthermore, we cannot control the quantity of QDs labeling with GLUT4, which is important for SPT experiments. What was worse, it was possible that QDs could be internalized nonspecifically by endocytosis. As a result, mechanism of GLUT4 translocation could not be observed by this method. In this study, we simplified the labeling procedure by using QDs secondary antibody conjugates and halted endocytosis by transferring L6-GLUT4myc cells to 4 °C when labeling QDs on GLUT4myc. The experimental time was significantly reduced, which reduced harm for live cells, and the complex of GLUT4myc and QDs was located on the PM at the beginning of imaging, which exposed whole dynamic process of GLUT4 internalization to us. We can adjust the reaction time and concentration of QDs to reduce quantity of QDs labeling with GLUT4 to make it more suitable for SPT experiments.

It is relatively easy to observe single QDs due to their greater brightness than other fluorescent probes. In addition, QDs provide extended periods of time for SPT experiments by reducing photobleaching [41]. In this study, we labeled GLUT4 by QDs in live L6 cells, observed internalization of GLUT4-QDs by z-scanning under confocal microscope. The exposure time of a single slice was 100 ms and the experiment lasted for half an hour, whereas conventional dyes could only be tracked for several seconds before photobleaching [34]. In fluorescent images, single QD could be detected easily. We can observe single GLUT4-QD in three-dimensional and investigate the detailed behavior of the dynamics of GLUT4. This method can reveal the entire dynamic process of GLUT4 translocation in live L6 cells in real time, which previous studies cannot do. Furthermore, temporal resolution can be improved by reducing spatial resolution and expose time to make the results more accurate. Future works can label GLUT4 with QDs and colocalize with cellular organelles by labeling them with bio-markers, such as EGFP-Rab11, EEA1 and TfR, to investigate the distribution of GLUT4 in an individual live L6 cell at different time. Furthermore, the dynamics of GLUT4 after its exit from the recycling endosome can be investigated to define the genesis of the specialized exocytic vesicles.

## 4. Conclusions

In this paper, we have developed a procedure for labeling GLUT4 using QDs and observed GLUT4 translocation in live L6 cells. We demonstrated that QDs can be targeted to GLUT4myc specifically and internalized through receptor-mediated endocytosis. In comparison with EGFP, QDs are easier to detect and extremely photostable for long term imaging. Combined with confocal microscopy, GLUT4-QDs translocation trajectories can be observed and analyzed in three-dimensional space for a remarkably long period of time. Based on this platform, tracking GLUT4 molecules in three-dimensional space and time can reveal unexpected details of GLUT4 cycling in live L6 cells. This approach opens new avenues for studying GLUT4 translocation in live cells.
